# Dissociative amnesia with fugue following a suicide attempt: a rare clinical intersection of identity loss and suicidal ambivalence: a case report

**DOI:** 10.3389/fpsyt.2026.1821981

**Published:** 2026-06-11

**Authors:** Mohammed Salah Alfahal, Mohamed Hassan, Maryam Alowais

**Affiliations:** Department of Psychiatry, Rashid Hospital, Dubai Health, Dubai, United Arab Emirates

**Keywords:** autobiographical memory, case report, dissociation, dissociative amnesia, dissociative fugue, identity loss, suicide attempt

## Abstract

**Background:**

Dissociative amnesia with dissociative fugue is a rare dissociative disorder characterized by abrupt loss of autobiographical memory and disruption of personal identity, most often emerging in the context of severe psychological stress. Although dissociative phenomena are increasingly recognized in individuals with suicidal behavior, the development of fugue states in the immediate aftermath of a suicide attempt remains rarely documented and poses significant diagnostic and therapeutic challenges.

**Case presentation:**

We report the case of a 50-year-old man with no documented psychiatric or neurological history who was admitted following a suicide attempt by bilateral wrist laceration. After medical stabilization, he exhibited profound retrograde autobiographical amnesia, including inability to recall his name, nationality, or personal life history, while semantic memory, language abilities, and general cognition remained largely preserved, as evidenced by fluent bilingual communication and intact broad factual knowledge. Comprehensive medical and neurological evaluation, including laboratory testing, toxicology screening, neuroimaging, and specialist consultations, excluded organic etiologies. During a three-week psychiatric admission, gradual recovery of selected autobiographical fragments occurred, although full identity restoration was not achieved at discharge. Management included supportive psychotherapy, safety monitoring, pharmacologic treatment for affective stabilization, and multidisciplinary psychosocial interventions.

**Discussion:**

This case illustrates a rare and clinically instructive intersection of suicidality and dissociative identity disruption. The pattern of selective retrograde autobiographical amnesia with intact semantic and procedural memory supports the retrieval inhibition model of dissociative amnesia, in which stress-related prefrontal inhibition functionally blocks access to identity-related memories without structural brain injury. An integrative framework combining neurobiological, cognitive, and psychodynamic perspectives suggests that dissociative fugue in this context may represent an extreme defensive response to overwhelming affective conflict, enabling psychological survival while temporarily disengaging identity from the intolerable internal experiences that precipitated the suicidal crisis.

**Conclusion:**

Dissociative amnesia with fugue should be considered when post-self-harm amnesia selectively impairs autobiographical memory despite preserved cognition and normal neurological findings. Accurate diagnosis requires longitudinal psychiatric assessment and exclusion of organic or feigned causes. No post-discharge follow-up data were available, which substantially limits prognostic conclusions. This case provides an educational framework for understanding the interaction between identity, memory, and self-preservation in acute psychiatric emergencies.

## Introduction

Dissociative amnesia is defined in the Diagnostic and Statistical Manual of Mental Disorders, Fifth Edition (DSM-5) as an inability to recall important autobiographical information, usually of a traumatic or stressful nature, that is inconsistent with ordinary forgetting (1). A severe subtype, dissociative amnesia with fugue, involves sudden and unexpected travel away from one’s customary environment accompanied by confusion about personal identity or inability to recall one’s past. Although rare, this condition presents substantial clinical challenges because of its dramatic presentation, potential for diagnostic confusion, and frequent association with psychological crises (2).

Contemporary neurobiological models conceptualize dissociative amnesia primarily as a disturbance of memory retrieval rather than structural brain injury. Functional neuroimaging studies implicate altered activity within the autobiographical memory network, particularly involving the hippocampus, medial temporal structures, and prefrontal cortical regions responsible for retrieval monitoring and inhibitory control (3, 4). These alterations are thought to disrupt access to episodic-autobiographical memory while preserving semantic and procedural knowledge, producing a characteristic dissociative pattern in which identity and personal life history are impaired despite intact general cognition (5). Clinically, episodes are frequently precipitated by overwhelming stress or psychological conflict, suggesting a stress-related modulation of memory systems.

Beyond neurobiology, cognitive and affective models describe dissociation as a maladaptive coping mechanism that temporarily attenuates intolerable emotional arousal by disrupting integration between memory, identity, and conscious awareness (5, 6). Such processes may reduce immediate psychological pain but can lead to profound disturbances in self-continuity and autobiographical coherence. This framework is particularly relevant in acute crises, where dissociative mechanisms may emerge as defensive responses to unbearable internal conflict (4).

A growing body of literature has highlighted the association between dissociative phenomena and suicidal behavior. Individuals who attempt suicide demonstrate significantly elevated dissociative symptoms compared with controls, as measured by validated instruments such as the Dissociative Experiences Scale and related measures (7). Similarly, patients diagnosed with dissociative disorders exhibit higher rates of repeated suicide attempts, suggesting that dissociation may influence emotional processing, risk perception, and behavioral control during self-harm episodes (8, 9). Psychodynamic perspectives further conceptualize dissociative states as reflecting suicidal ambivalence, wherein psychological withdrawal or identity disruption functions as an unconscious alternative to irreversible self-destruction (10, 11). Despite these theoretical links, cases in which dissociative fugue develops immediately after a suicide attempt remain rarely documented.

Diagnostically, dissociative amnesia with fugue is a diagnosis of exclusion. Careful evaluation is required to differentiate it from neurological conditions, substance-related states, neurocognitive disorders, psychotic illnesses, and potential volitional feigning, particularly in medicolegal contexts (12). Because no single biomarker exists, diagnosis relies on longitudinal clinical observation, exclusion of organic pathology, and integration of psychological and behavioral findings.

The present case report describes a patient admitted following a suicide attempt who subsequently presented with profound autobiographical identity loss consistent with dissociative amnesia with fugue. Notably, the patient retained partial memory of the suicidal act while remaining amnestic for his personal identity and life history, illustrating a selective disruption of autobiographical memory. By integrating neurobiological, cognitive, and psychodynamic perspectives, this case aims to provide an educational clinical framework for understanding the complex interaction between dissociation and suicidality and to contribute to the limited literature describing dissociative fugue in the acute aftermath of self-harm.

## Case presentation

### Patient information

A 50-year-old man with no available collateral history and no documented psychiatric or medical diagnoses was brought to the emergency department in late April 2025 after being found in a public area in Dubai with bilateral self-inflicted wrist lacerations and a handwritten suicide note in his possession. Based on his fluency in English and Farsi and limited Arabic vocabulary, he appeared to be a non-Arabic-speaking expatriate, likely residing and working in the UAE. A suicide note referencing financial stressors was found with him, suggesting he had been employed or engaged in financial activity prior to admission, though his precise occupational and living circumstances could not be verified. The patient denied alcohol or illicit substance use, and no family psychiatric history could be established. Premorbid functioning, developmental history, trauma exposure, and psychosocial supports could not be verified because of the absence of informants and the patient’s profound autobiographical memory impairment at presentation. The unavailability of collateral history represents a significant source of residual diagnostic and contextual uncertainty throughout this report.

### Presenting concerns

The patient presented following an apparent suicide attempt by wrist cutting. During initial psychiatric assessment, he reported extensive gaps in autobiographical memory, including inability to recall his name, age, nationality, place of residence, relatives, or personal life history. He was unable to provide any contact information or identifying details.

### Clinical findings

The patient’s clinical course, investigations, and interventions across the three-week admission are summarized in **Table 1**.

**Table 1:** Timeline of clinical course.

Hospital day/date (2025)	Clinical status & key findings	Diagnostic evaluation	Interventions & clinical outcome
Day 0 (Late Apr)	Found in public area with bilateral wrist lacerations and suicide note; profound autobiographical amnesia including loss of identity; medically stable after initial care	Emergency laboratory workup; CT brain showed chronic small-vessel changes without acute pathology; toxicology negative	Surgical repair of wrist wounds (R 21 sutures, L 17 sutures); correction of hypokalemia; admitted to psychiatry; initiated sertraline 50 mg daily and short-term lorazepam; high-level suicide observation
Days 1–4	Persistent identity amnesia; recalls sensory fragments of self-harm but not antecedent planning; affective flattening and “blank mind” described; no psychosis	Neurology and internal medicine consultations excluded neurological or metabolic causes	Lorazepam gradually tapered; supportive psychotherapy initiated; orientation and reassurance strategies employed
Days 5–10	Partial recovery of semantic and autobiographical fragments (e.g., sports, music preferences); continued inability to identify self; reported vague financial stressors	Ongoing longitudinal psychiatric assessment supporting dissociative etiology	Lorazepam discontinued due to sedation; continuation of sertraline; antihypertensive therapy initiated for persistent elevated blood pressure
Days 11–14	COVID-19 infection confirmed; psychiatric status stable with no behavioral deterioration; autobiographical deficits unchanged	Medical monitoring during isolation	Supportive respiratory care; continuation of psychiatric treatment and observation
Days 15–21	Gradual emotional stabilization; persistent identity amnesia with preserved semantic cognition; denies suicidal ideation; improved insight into condition	Final multidisciplinary diagnostic synthesis supporting dissociative amnesia with fugue	Observation level reduced; social work and embassy liaison initiated for identity verification; discharge planning completed
Discharge (≈Week 3)	Psychiatrically stable; partial autobiographical recovery; full identity not restored	—	Discharged with outpatient psychiatric follow-up, continuation of sertraline and medical treatment, and psychosocial reintegration planning

#### Medical stabilization and physical examination

On arrival, the patient was medically stabilized following management of bilateral wrist lacerations (right wrist: 21 sutures; left wrist: 17 sutures). Internal medicine and neurology evaluations confirmed medical stability for psychiatric admission. Initial vital signs revealed marked hypertension, which improved with treatment during admission.

#### Mental status examination and neuropsychiatric profile

Serial psychiatric assessments consistently demonstrated a calm, cooperative, and communicative patient. Speech was fluent and coherent, with normal rate and prosody. Thought processes remained logical and goal-directed, with no evidence of formal thought disorder. He repeatedly denied hallucinations, delusions, or ongoing suicidal or homicidal intent following admission.

Affect ranged from mildly constricted to euthymic. The patient frequently described a subjective experience of mental “blankness, “ accompanied by feelings of boredom and diminished personal purpose, while also expressing regret regarding the self-harm act.

The defining clinical feature was severe retrograde autobiographical amnesia, predominantly affecting identity and personal life history, in the context of preserved semantic knowledge and intact general cognition. Serial clinical assessments evaluated multiple cognitive domains. Orientation to time and place was confirmed intact across repeated evaluations, whereas orientation to personal identity remained markedly impaired throughout the admission. Attention and working memory were assessed clinically and found intact, as evidenced by the patient’s ability to sustain coherent and goal-directed conversation, follow multi-step instructions, and maintain consistent logical discourse without distractibility or perseveration. Language function was demonstrably preserved across two languages (English and Farsi). Broad semantic knowledge, including intact recall of geography, current affairs, and cultural information, was evidenced throughout clinical interviews. Executive functioning was assessed through the patient’s capacity for goal-directed reasoning and communication, which remained unimpaired. Procedural memory was intact, as evidenced by preserved self-care skills and activities of daily living. He knew only a few words in Arabic, further supporting relative preservation of semantic and procedural memory networks. It is important to acknowledge that no formal standardized bedside cognitive screening instrument or neuropsychological battery (e.g., Montreal Cognitive Assessment) was administered during this admission. The characterization of preserved cognition therefore rests on repeated clinical and behavioral observations across the multidisciplinary team rather than psychometric data, a limitation discussed further in the Strengths and Limitations section.

### Phenomenology of the suicidal act and memory pattern

The patient described fragmented recall of events surrounding the self-harm episode. He recalled visiting a shopping area and subsequently traveling to a waterfront location during early morning hours while carrying a small bag containing water, food items, and a cutting instrument. He remembered sensory and emotional aspects of the self-harm act itself, including the sound of the blade and immediate regret; however, he could not recall antecedent emotional states, planning processes, or writing the suicide note.

Police reports indicated that the note referenced financial stressors. During later interviews, the patient suggested possible financial or legal difficulties, although his account remained fragmented and inconsistent due to ongoing amnesia.

### Diagnostic assessment

Comprehensive multidisciplinary evaluation was undertaken to determine the etiology of the severe autobiographical memory disturbance.

### Laboratory and medical investigations

Initial laboratory testing was unremarkable except for transient hypokalemia, which was corrected. Other biochemical parameters were within normal limits. Toxicology screening did not reveal evidence of intoxication or overdose by commonly assessed substances.

### Neuroimaging and medical consultation

Computed tomography (CT) of the brain demonstrated chronic periventricular white-matter hypodensities consistent with mild small-vessel ischemic changes, without acute intracranial pathology. Neurological and internal medicine evaluations found no evidence of structural, epileptic, metabolic, or neurodegenerative pathology that could explain the degree or selectivity of the amnesia. The patient was therefore considered neurologically stable and appropriate for psychiatric management. Magnetic resonance imaging (MRI) of the brain and electroencephalography (EEG) were not performed during this admission. MRI was not pursued as the neurological consultation identified no clinical indicators of an acute structural lesion, and the CT findings were limited to chronic nonspecific small-vessel changes. EEG was not obtained, as the clinical presentation lacked features suggestive of ictal or post-ictal states, including no witnessed seizure activity, no stereotyped automatisms, no postictal confusion, and no fluctuating level of consciousness. The authors acknowledge that the absence of MRI and EEG represents a significant methodological limitation. MRI would have provided greater sensitivity for subtle structural changes, hippocampal pathology, or demyelinating lesions not visible on CT. EEG would have more definitively excluded subclinical epileptic activity, particularly temporal lobe epilepsy, as a contributing factor. These limitations are further addressed in the Strengths and Limitations section.

### Psychiatric diagnostic evaluation

Longitudinal psychiatric observation revealed persistent retrograde autobiographical amnesia involving identity and life history, with preservation of semantic memory, procedural skills, language function, and executive abilities. No psychotic symptoms or global cognitive deficits were observed.

The differential diagnosis included:

- Dissociative amnesia with dissociative fugue (primary working diagnosis): supported by abrupt identity loss, preserved semantic cognition, and confirmed wandering behavior, and absence of organic causes. The DSM-5 fugue specifier (300.13/F44.1) requires confused wandering or bewildered travel alongside identity confusion, and is distinguished from uncomplicated dissociative amnesia by the additional behavioral element of purposeful, unrecollected travel. In this case, three elements support the fugue specifier. First, the patient was found at a public waterfront location distant from any known place of residence or routine activity, having traveled there in the early morning hours while carrying a bag of provisions, constituting unrecollected and purposeful travel. Second, upon discovery he was entirely unable to identify himself, explain his presence, or account for his journey, fulfilling the criterion of bewildered travel accompanied by identity confusion. Third, these behavioral elements extend the presentation beyond simple dissociative amnesia and are not explained by organic or substance-related pathology.
- Major depressive disorder or adjustment-related disturbance: considered given the suicidal behavior and reported stressors, though full syndromal assessment was limited by amnesia and lack of collateral information.
- Psychotic disorders: considered unlikely due to absence of hallucinations, delusions, or disorganized thinking across repeated evaluations.
- Neurological or neurocognitive amnesia: excluded through neuroimaging and specialist consultation.
- Malingering or feigned amnesia: considered given potential psychosocial stressors but judged unlikely based on multiple converging lines of clinical evidence. The patient’s symptom profile remained internally consistent across independent encounters with all members of the multidisciplinary team. He demonstrated genuine and observable distress related to his amnesia, including expressed frustration, existential concern, and emotional discomfort when confronted with the absence of personal identity. Memory recovery occurred gradually and spontaneously, following a clinically plausible trajectory, with earlier return of semantic and procedural knowledge preceding partial restoration of autobiographical fragments, a pattern consistent with known recovery profiles in dissociative amnesia. No external incentive for amnesia simulation was identified; there were no apparent legal proceedings, immigration claims, or financial benefits that would have been served by feigning. The absence of a contradictory clinical pattern across extended observation further reduced the likelihood of volitional simulation.
- Ganser Syndrome: considered given the dissociative amnesia and potential medicolegal context. However, Ganser Syndrome characteristically features approximate or near-miss answers (Vorbeireden), which were entirely absent in this patient, who responded accurately when able and acknowledged gaps rather than providing approximate responses. This clinical distinction, combined with the absence of any identifiable secondary gain, supported exclusion of this diagnosis.
- Dissociative Identity Disorder (DID): considered given the profound identity disruption. However, DID is characterized by the presence of two or more distinct personality states with recurrent amnesia between them, typically associated with a history of severe early trauma. In this patient, no evidence of alternate identity states, switching behavior, or amnesia for distinct identity episodes was observed across repeated evaluations. The clinical presentation was consistent with a single, contiguous episode of retrograde autobiographical amnesia rather than the recurrent, inter-identity amnesia characteristic of DID.

Based on multidisciplinary assessment and longitudinal clinical observation, the final diagnosis was dissociative amnesia with dissociative fugue. A concurrent depressive episode was considered clinically likely given the suicidal behavior and evident psychosocial stressors, though formal syndromal diagnosis could not be established due to ongoing autobiographical amnesia and absence of collateral history. It should be noted that no standardized dissociation rating instrument, such as the Dissociative Experiences Scale (DES), was administered during this admission. Formal dissociation assessment would have provided a quantified characterization of dissociative severity and further strengthened the diagnostic formulation; this is acknowledged as a limitation discussed in the Strengths and Limitations section.

### Therapeutic interventions

#### Medical management

Medical care included surgical repair of wrist wounds, correction of electrolyte imbalance, and initiation of antihypertensive therapy (amlodipine, valsartan, and bisoprolol) for persistent blood pressure elevation. During hospitalization, the patient developed COVID-19 infection requiring temporary isolation and supportive respiratory care, without psychiatric destabilization.

#### Psychiatric management

Pharmacological treatment consisted of:

- Sertraline 50 mg daily, initiated for mood stabilization and anxiety-related symptoms.
- Lorazepam 1 mg twice daily, prescribed briefly for anxiety and behavioral stabilization, then tapered and discontinued due to sedation and dizziness.

Psychotherapeutic management emphasized supportive psychotherapy, emotional containment, reassurance, and orientation strategies. Attempts at forced or confrontational memory retrieval were intentionally avoided. Nursing observation levels were gradually reduced as acute suicide risk diminished.

2 Psychosocial interventions

Given ongoing identity uncertainty, social work teams coordinated identity verification procedures through institutional and embassy liaison pathways. Management required close collaboration between psychiatry, nursing, neurology, internal medicine, and social services.

3 Follow-up and outcomes

The patient remained hospitalized for approximately three weeks. During admission, partial recovery of autobiographical fragments occurred, including recollection of personal preferences (e.g., football affiliation, music interests) and possible elements of his name. However, full recovery of identity and personal history was not achieved prior to discharge.

Clinically, the patient demonstrated sustained behavioral stability, resolution of acute suicidality, preserved reality testing, and improved emotional regulation. At discharge, he remained partially amnestic but expressed curiosity and motivation to recover his identity. The personality fragments that emerged during recovery offered a limited but meaningful window into the patient’s premorbid character. His strong identification with a specific football club, his preferences for particular musical styles, and his expressed sense of humor during interactions with nursing staff suggested a sociable and culturally engaged individual. His distress at being unable to name or describe himself, and his sustained motivation to search for clues about his identity, reflected preserved self-awareness and personal agency despite the amnestic state. These recovered fragments informed therapeutic rapport-building and guided the clinical team’s approach to psychosocial reintegration. Discharge planning included continuation of sertraline and antihypertensive medications, outpatient psychiatric review within two weeks of discharge, ongoing social work involvement for identity documentation and reintegration support, and referral for structured trauma-informed psychotherapy once autobiographical continuity was sufficiently stabilized. The clinical team also documented guidance for emergency re-presentation should suicidal ideation re-emerge as autobiographical memory recovered. It is important to note that no post-discharge follow-up data are available for this patient. Contact could not be maintained following discharge, partly due to the incomplete identity verification at the time of release. This represents a significant limitation of the report, as recovery trajectory and long-term outcome are central to understanding the natural history of dissociative fugue. The absence of longer-term outcome data prevents reliable conclusions about the prognosis of this individual case and is discussed further in the Strengths and Limitations section.

## Discussion

Dissociative amnesia with fugue is among the rarest dissociative phenomena, with lifetime prevalence estimates around 0.2%, and remains underrepresented in the literature because most evidence derives from individual case reports rather than systematic studies (2). Despite its rarity, the condition carries substantial clinical importance owing to diagnostic uncertainty, frequent misclassification as neurological or malingering presentations, and its documented association with severe psychological distress and suicidal behavior. The present case illustrates a particularly uncommon clinical sequence in which profound autobiographical amnesia emerged immediately following a suicide attempt, offering an opportunity to explore the interplay between dissociation, identity disruption, and suicidal ambivalence.

Current neurobiological models conceptualize dissociative amnesia as a disorder of memory retrieval rather than memory storage or structural brain injury. Autobiographical memory depends on coordinated activity across hippocampal structures, medial temporal regions, and prefrontal cortical networks responsible for self-referential processing, emotional regulation, and executive control of recall. Functional neuroimaging studies demonstrate reduced activation of these networks during attempts to retrieve autobiographical memories in patients with dissociative amnesia, suggesting functional inhibition rather than irreversible damage (3, 4). The hippocampus plays a central role in contextual integration of personal experiences, while prefrontal regions regulate access to emotionally salient memories. Disruption of connectivity between these regions may result in selective impairment of autobiographical recall with preservation of semantic knowledge and procedural abilities (4). This mechanism closely mirrors the clinical pattern observed in our patient, who retained language abilities, general knowledge, and cultural information while remaining unable to access identity-related memories.

Bridging neurobiology and clinical presentation, cognitive frameworks propose that dissociative amnesia arises from stress-induced inhibition of memory retrieval processes. The retrieval inhibition hypothesis, as elaborated by Markowitsch and Staniloiu (13), proposes that overwhelming emotional stress activates prefrontal inhibitory mechanisms that suppress access to traumatic or conflict-laden autobiographical material. Rather than reflecting memory loss or structural damage, the amnesia represents an active functional blockade that protects the individual from intolerable affective states. In the present case, the patient’s repeated description of his mind as “blank” and his inability to comprehend the motivations behind his own self-harmful actions directly mirrors this model: the very material most closely linked to intolerable conflict (personal identity, planning, motivation) was selectively blocked, while emotionally neutral semantic content remained accessible. This selective inhibitory gradient is precisely what the retrieval inhibition hypothesis would predict when autobiographical-emotional networks are under maximal stress-related suppression.

In the present case, the severe psychological context surrounding the suicide attempt may have precipitated such inhibitory processes, resulting in abrupt loss of autobiographical continuity while sparing semantic cognition. The patient’s repeated description of his mind as “blank” and inability to understand the motivations behind his own actions aligns with this model, reflecting disconnection between emotional memory and conscious self-awareness (5).

From a psychodynamic perspective, dissociative fugue has historically been conceptualized as a symbolic escape from unbearable psychological conflict. Classical authors proposed that fugue states may function as unconscious alternatives to suicide, enabling psychological withdrawal without physical death (11, 14). Contemporary formulations interpret such dissociative phenomena within Schneidman’s model of suicidal ambivalence, in which the wish to live and wish to die coexist in dynamic tension (10).

Our patient’s presentation is consistent with this conceptual framework of suicidal ambivalence, though this interpretation should be regarded as a plausible hypothesis applied to the observable clinical features rather than a confirmed mechanistic conclusion. The handwritten suicide note found on his person is itself a significant and clinically underexplored element in this regard. The very act of writing and carrying a note directed at others implies a residual communicative impulse — a reaching toward others — that coexists with the self-destructive act. The note’s reference to financial stressors further suggests a wish to be understood, perhaps even helped, rather than a purely lethal intent. This communicative gesture, followed by the subsequent fugue-state erasure of the note’s authorship from conscious awareness, powerfully illustrates the ambivalence at the heart of the presentation: the patient simultaneously produced evidence of his identity and inner world, then lost access to both. While he engaged in deliberate self-harm, he described immediate regret and emotional disgust during the act, followed by subsequent loss of identity and autobiographical memory. Within this framework, dissociation may plausibly represent a form of defensive psychological reorganization that temporarily removes awareness of unbearable conflict, allowing survival while postponing confrontation with distressing internal states, a hypothesis consistent with this case but requiring replication across larger clinical samples (9).

The association between dissociation and suicidal behavior is increasingly supported by empirical evidence. Individuals who attempt suicide show higher levels of dissociative symptoms than matched psychiatric controls, as measured by standardized instruments such as the Dissociative Experiences Scale (7). Furthermore, dissociative disorders are associated with significantly higher rates of repeated suicide attempts (8). Dissociation may reduce emotional pain and fear, temporarily lowering the threshold for self-harm by blunting conscious awareness of intent and consequences (9).

In this case, the selective nature of memory impairment is notable. The patient retained sensory fragments of the suicidal act including the sound of the blade and emotional reactions yet remained amnestic for antecedent planning and personal identity. This selective dissociation reinforces prior observations that emotionally intense episodic memories may be fragmented rather than completely erased, while semantic memory remains largely intact (2).

Dissociative fugue remains a diagnosis of exclusion and requires careful differentiation from neurological, substance-related, and volitional causes of amnesia (2, 9). Organic etiologies including temporal lobe epilepsy, traumatic brain injury, intoxication, transient global amnesia, and neurodegenerative disorders must be systematically excluded through clinical assessment, laboratory investigations, neuroimaging, and specialist consultation. In the present case, condition-specific reasoning supported the exclusion of each major organic alternative. Transient global amnesia (TGA) was considered unlikely, as TGA characteristically resolves within 24 hours, predominantly affects anterograde memory, and presents with repetitive questioning and disorientation rather than the sustained, selective, retrograde autobiographical loss observed here over three weeks. Temporal lobe epilepsy (TLE) was excluded on clinical grounds: there were no witnessed seizure events, automatisms, postictal confusion, or fluctuating consciousness, and neurological consultation did not identify any clinical features suggestive of an epileptic process. Delirium was excluded based on serial mental status evaluations confirming consistent alertness, sustained attention, coherent communication, and absence of perceptual disturbances or fluctuating consciousness; laboratory investigations revealed no metabolic derangements beyond a transient, corrected hypokalemia. Evolving neurocognitive disorder was considered given the patient’s age and the CT finding of chronic small-vessel changes; however, the onset was abrupt rather than insidious, global cognitive functions were preserved, and no behavioral features typical of neurocognitive disorders were observed, findings inconsistent with this diagnosis. Substance-related amnesia was excluded by negative toxicology screening and absence of clinical signs of intoxication or withdrawal. Internal medicine and neurology evaluations found no evidence of structural, epileptic, or metabolic causes, and neuroimaging demonstrated only nonspecific chronic small-vessel changes.

Malingering represents an important differential diagnosis, particularly in cases involving potential legal or financial consequences. Features suggestive of malingering include inconsistent symptom reporting, external incentives, and absence of spontaneous memory fragments (12). In the present case, several converging lines of clinical evidence supported the conclusion that deliberate feigning was unlikely. The patient’s symptom profile remained internally consistent across multiple independent encounters with different members of the multidisciplinary team, including psychiatrists, nurses, neurologists, and social workers. He demonstrated genuine and observable distress related to his amnesia, including expressed frustration, existential concern, and emotional discomfort when confronted with his lack of personal identity, behavioral features inconsistent with volitional symptom production. Memory recovery occurred gradually and spontaneously, following a clinically plausible trajectory, with earlier return of semantic and procedural knowledge preceding partial restoration of autobiographical fragments, consistent with known patterns in dissociative amnesia. No external incentive for feigning was identified; there were no apparent legal proceedings, immigration claims, or financial benefits that would have been served by sustained amnesia simulation. The consistency of presentation across extended multidisciplinary observation, combined with spontaneous and partial memory recovery, further strengthened diagnostic confidence.

Management of dissociative fugue centers on safety stabilization, supportive psychotherapy, and treatment of comorbid symptoms. There is no pharmacological intervention proven to directly reverse dissociative amnesia; medications are typically used to address associated depressive, anxiety, or sleep-related symptoms (15). In this case, sertraline was initiated to support affective stabilization, while lorazepam was used briefly and discontinued due to sedation. Supportive psychotherapy focused on orientation, reassurance, emotional containment, and avoidance of coercive memory recovery attempts, consistent with recommended clinical practice.

The multidisciplinary approach was essential, involving psychiatry, neurology, nursing staff, and social services. Observation levels were gradually reduced as acute suicide risk subsided, while social work and embassy involvement addressed identity verification and discharge planning. This highlights the practical importance of psychosocial and medico-legal considerations in cases of identity loss.

The clinical course of dissociative fugue varies widely. Some patients recover autobiographical memory rapidly, while others remain amnestic for months or even years (2). At discharge, the patient remained unable to recall his full identity but had regained partial semantic and autobiographical fragments, suggesting gradual reintegration rather than abrupt recovery. Given that recovery in dissociative amnesia with fugue may extend over months to years, the absence of post-discharge follow-up in this case substantially limits prognostic inference and prevents reliable conclusions about the longer-term trajectory of memory recovery. Persistent amnesia may lead to significant functional impairment and psychosocial vulnerability, underscoring the need for long-term follow-up, rehabilitation, and social support, particularly when ongoing stressors such as financial difficulties remain unresolved (16).

This case supports an integrative understanding of dissociative fugue as the convergence of neurobiological, cognitive, and psychodynamic processes. Neurobiologically, acute suicidal stress may have activated prefrontal inhibitory circuits that suppressed retrieval across the hippocampal-prefrontal autobiographical memory network, functionally blocking access to identity-related information while sparing semantic and procedural systems (4, 13). Psychologically, this same overwhelming affective state may have simultaneously triggered a psychodynamic defensive dissociation, severing conscious access to the intolerable internal conflict that preceded the attempt (10, 15). Cognitively, the resulting disruption of autobiographical integration impaired continuity of self in the very domain where the conflict resided (9). What distinguishes this integrative account from a simple enumeration of mechanisms is the proposed convergence: neurobiological inhibition and psychodynamic defense may not be independent but rather two descriptions of the same stress-response process operating at different levels of analysis. Together, these mechanisms may allow short-term psychological survival at the cost of identity coherence, a trade-off that this case illustrates with unusual clinical clarity.

Clinicians should consider dissociative amnesia with fugue in patients presenting with post-suicidal amnesia, especially when autobiographical memory loss occurs in the presence of preserved semantic cognition and normal neurological findings (9, 15). Careful longitudinal assessment is critical, as suicidality may fluctuate with memory recovery: as autobiographical continuity is restored, the affective conflict that precipitated the crisis may re-emerge, and suicide risk should be continuously reassessed throughout this process. Future research should focus on longitudinal neurocognitive studies, standardized assessment tools, and functional neuroimaging to better clarify mechanisms of memory recovery and identify predictors of prognosis.

Overall, this case demonstrates how dissociative fugue may emerge at the intersection of overwhelming stress, suicidal ambivalence, and disrupted autobiographical memory processing. Recognition of this phenomenon is essential to avoid misdiagnosis, to provide compassionate and supportive care, and to improve understanding of the complex relationship between identity, memory, and self-preservation in psychiatric crisis.

## Strengths and limitations

This report contributes to the literature by documenting a prolonged fugue state directly following a suicide attempt, with comprehensive multidisciplinary evaluation and longitudinal observation. The main limitations include the following. First, no formal standardized cognitive screening instrument or neuropsychological battery was administered; the characterization of preserved cognition is therefore based on clinical and behavioral observations rather than psychometric data, and future cases should include formal assessment as standard practice. Second, advanced neuroimaging (MRI) and electroencephalography (EEG) were not performed; while clinical and CT evaluation excluded acute organic pathology, MRI and EEG would have provided greater sensitivity for subtle structural or epileptic contributions. Third, collateral history was entirely unavailable, rendering premorbid functioning, trauma exposure, and psychosocial context unverifiable; psychodynamic interpretations advanced in the discussion should accordingly be understood as plausible hypotheses rather than established conclusions. Fourth, no post-discharge follow-up data are available, as contact could not be maintained following discharge partly due to incomplete identity verification; this substantially limits any prognostic conclusions about the trajectory of memory recovery in this case. Fifth, no standardized dissociation rating instrument, such as the Dissociative Experiences Scale (DES), was administered during this admission; formal dissociation assessment would have provided an objective, quantified characterization of dissociative severity and symptom profile, and would have further strengthened the diagnostic formulation. Future case reports in this area should incorporate the DES or an equivalent instrument as a routine component of the diagnostic workup.

## Conclusion

This case describes a rare presentation of dissociative amnesia with fugue emerging immediately after a suicide attempt, illustrating the complex interaction between severe psychological stress, dissociative mechanisms, and suicidal ambivalence. The case emphasizes the importance of maintaining a high index of suspicion for dissociative disorders when patients present with post-suicidal amnesia, particularly when autobiographical memory loss occurs in the context of preserved semantic cognition and normal neurological evaluation. Comprehensive diagnostic assessment—including exclusion of organic causes and careful longitudinal psychiatric observation—is essential for accurate diagnosis and appropriate management. Supportive, non-confrontational psychiatric care remains central to treatment, with attention to safety, emotional containment, and gradual reintegration of identity. Ultimately, this case underscores how dissociative fugue may function as an extreme psychological response to intolerable stress, offering temporary psychological escape at the cost of autobiographical continuity. The principal clinical learning points from this case are summarized in **Table 2**.

**Table 2:** Learning points from case report.

Learning point	Key concept	Clinical implication
A Crisis of Self	Dissociative amnesia with fugue can represent a catastrophic but life-preserving disintegration of identity in response to unbearable stress.	View the fugue not just as a symptom, but as a profound defensive operation. The therapeutic goal is to provide a safe environment for the gradual reintegration of the self, without forceful confrontation.
A Diagnosis of Exclusion	It requires rigorous medical and neurological workup to rule out organicity and careful clinical assessment to distinguish from malingering.	Adhere to a strict diagnostic protocol including neuroimaging, toxicology screens, and neurology consultation. Document the patient’s consistency, lack of secondary gain, and genuine distress to support the diagnosis.
Dynamic Risk	The fugue state may temporarily lower acute suicide risk, but this risk must be continuously re-evaluated as memory returns and underlying conflicts re-emerge.	Suicide risk is not static. Implement close monitoring throughout hospitalization, especially during memory recovery. Discharge planning must address the precipitating stressors to prevent relapse.
Integrated Care is Paramount	Management extends beyond medication to include supportive psychotherapy, safety monitoring, and practical social reintegration.	Employ a multidisciplinary team (psychiatry, nursing, social work). Focus on reassurance, orientation, and affective containment in therapy, while actively working on real-world issues like identity and housing.
Neurobiological Correlation	The clinical presentation of selective autobiographical amnesia is strongly correlated with dysfunction in the hippocampal-prefrontal autobiographical memory network.	When neuroimaging is normal and cognitive profile shows selective autobiographical impairment with preserved semantics, consider functional rather than structural amnesia. In research or specialist settings, consider referral for functional neuroimaging (fMRI) to objectively characterize memory network disruption and monitor recovery trajectory. Document cognitive domains systematically across admissions to build an evidence base for the selectivity claim.

## Patient perspective

The patient described profound regret regarding the suicide attempt, characterizing it as a mistake and expressing relief at survival. He reported significant frustration and distress related to his inability to remember his name or personal identity, repeatedly describing his mind as feeling “blank.” Despite this experience, he demonstrated curiosity about the world around him and responded positively to meaningful cues such as music and football, which appeared to evoke emotional familiarity. He consistently denied ongoing suicidal intent and expressed a desire to recover his identity, describing the physical pain and emotional consequences of the attempt as reasons he would not repeat such an act.

## Data Availability

The original contributions presented in the study are included in the article/supplementary material. Further inquiries can be directed to the corresponding author.
